# Vagal nerve signals are modulated by spontaneous seizures in Genetic Absence Epilepsy Rats from Strasbourg

**DOI:** 10.3389/fnins.2025.1568261

**Published:** 2025-07-07

**Authors:** Elise Collard, Enrique Germany Morrison, Elena Acedo Reina, Ayse S. Dereli, Auriane Apaire, Antoine Nonclercq, Riem El Tahry

**Affiliations:** ^1^Clinical Neuroscience, Institute of Neuroscience (IoNS), Université Catholique de Louvain, Brussels, Belgium; ^2^BEAMS Department, Université Libre de Bruxelles, Brussels, Belgium; ^3^Department of Neurology, Center for Refractory Epilepsy, Cliniques UniversitairesSaint-Luc, Brussels, Belgium; ^4^Walloon Excellence in Life Sciences and Biotechnology (WELBIO) Department, WELResearch Institute, Wavre, Belgium

**Keywords:** vagus nerve activity (VNA), GAERS (Genetic Absence Epilepsy Rats from Strasbourg), absence seizure, autonomic dysfunction, parasympathetic, NREM (non REM) sleep, age

## Abstract

**Introduction:**

One-third of epileptic patients are resistant to conventional treatments. Vagus nerve stimulation is a promising therapy, especially when applied early during seizure onset. This study explores vagus nerve activity (VNA) during seizures in Genetic Absence Epilepsy Rat from Strasbourg (GAERS) model and explores how VNA changes with epilepsy duration.

**Methods:**

Eleven rats (4, 6, and 10 months old, *n = 4*, 4, 3, respectively) were continuously recorded with electroencephalography, VNA recordings, and video for 24 h. Ictal VNA root mean square (RMS) values preceded by NREM sleep extracted from 11 rats were studied in a total of 620 seizures.

**Results:**

Overall, VNA RMS increased during seizures, with a median rise of 60%. However, this modulation decreased with age, despite stable seizure severity. Significant differences in VNA activity and inter-quartile range were observed between age groups.

**Discussion:**

These results support seizure severity-dependent changes in ictal VNA modulation and point toward the potential of VNA as a biomarker for seizure detection and autonomic dysfunction.

## Highlights

• Vagus nerve activity (VNA) recording increases during absences in GAERS.
• Ictal VNA response decreases with age despite stable epilepsy.
• Individual variability of the ictal VNA modifications decreases with age.

## 1 Introduction

Epilepsy is a neurological disorder marked by recurrent seizures, leading to neurobiological, cognitive, psychological, and social consequences, accompanied by stigma, comorbidities, and high economic costs (Beghi, 2020). Vagus Nerve Stimulation (VNS) is a treatment used for patients with refractory epilepsy (Afra et al., 2021; Ben-Menachem, 2002). VNS consists of an implantable pulse generator that delivers electrical pulses in continuous programmable ON/OFF cycles. VNS response varies, with a 50% seizure reduction in half of the implanted patients (Elliott et al., 2011). However, patient phenotype, epilepsy type or etiology do not predict treatment response (Englot et al., 2016; Labar, 2004). Studies suggest that on-demand VNS at seizure onset can reduce seizure duration or intensity (McLachlan, 1993; Zabara, 1992). The VNS aspire model uses a heart-based seizure detection algorithm to deliver on-demand VNS in addition to continuous stimulation in duty cycles (Boon et al., 2001). However, many patients do not exhibit ictal tachycardia during seizures and exhibit other sympathetic responses, such as increased respiration or blood pressure. Additionally, from infancy to adulthood, tonic-clonic and focal seizures can activate sympathetic responses or lead to parasympathetic dominance, affecting peristalsis, pupil dilation, and heart or respiratory rates (Devinsky, 2004). The vagus nerve, integral part of the parasympathetic system, helps balance sympathetic system dysregulation (Teckentrup and Kroemer, 2024). Thus, developing seizure detection based on parasympathetic modulation during a seizure seems promising.

Seizures affect autonomic function during, after, and between seizures (Devinsky, 2004) in many types of chronic epilepsy, including idiopathic generalized epilepsy (IGE) including childhood absence epilepsy (CAE), a common form of pediatric epilepsy that is typically non-eligible for surgery. Absence seizures are the semiology of several other epilepsy syndromes, such as juvenile absence epilepsy, juvenile myoclonic epilepsy, and myoclonic absence epilepsy (Crunelli and Leresche, 2002; Smyk and van Luijtelaar, 2020). The GAERS model established in 1982 closely mirrors the characteristics of IGE showing similar behavioral and electrophysiological traits to human absence epilepsy, and responds to antiseizure drugs closely parallels clinical observations (Depaulis et al., 2016). Literature suggests that abnormal autonomic regulatory functions are disrupted by epilepsy in TLE and IGE (Shaker et al., 2021) with cases of ictal tachypnea and bradypnea during CAE (Jansen et al., 2013). This makes the GAERS model an interesting tool for studying autonomic changes during seizures.

Autonomic dysfunction in epilepsy—marked by increased sympathetic tone and reduced HRV—can result from chronic seizures, antiseizure treatments, or epilepsy itself (Thijs et al., 2021). Reduced HRV has been observed during interictal discharges in absence epilepsy (Pradhan et al., 2011) and in WAG/Rij rats, a genetic model of absence epilepsy, where aging leads to diminished HRV and increased sympathetic dominance (Mamalyga and Mamalyga, 2019). These findings highlight the interest in studying VNA after an accumulation of seizures to better understand autonomic involvement over time.

We aim to study whether VNA modifications occur during absence seizures in the GAERS model. We hypothesize that autonomic ictal activation during absences can be recorded through continuous VNA recording. Additionally, we hypothesize that ictal VNA modifications will be less prominent with increasing age and cumulative occurrence of absences.

## 2 Materials and methods

### 2.1 Animals

All experimental protocols were approved by Brussels Environment ethical committee (2023/UCL/MD/09) and complied with EU directive 2010/63/EU. Female and male GAERS rats [4, 6, and 10 months (*n* = 4/age)] bred in-house under the License LA 1230664 from a Grenoble Institute of Neuroscience (France) parental stock, was used. Animals were housed in groups of 2–3 under standard conditions (12 h light cycle, 23–24°C, 45%–55% humidity) with *ad libitum* food and water, and provided with enrichment materials.

### 2.2 Experimental procedures

Twelve rats were used and recorded (VNA, EEG, and video) for 48 h. The 2 days EEG recordings were used to characterize seizures across the different age groups. Only the first 24 h per rat were used for the VNA analysis.

### 2.3 Surgical procedures

Anesthesia was induced with 5% sevoflurane in O_2_ and maintained at 2.5% (3 L/min, Dräger-Vapor2000). Tradamol^®^ (5 mg/kg, s.c.) was given 30 min pre-surgery. Body temperature was maintained at 37°C. Craniotomies were performed with a hand drill, and five stainless-steel screws were implanted for epidural EEG recordings at standard coordinates. To implant the vagus nerve electrode, the left cervical vagus nerve was exposed via blunt dissection. A custom 3-lead micro-cuff electrode (300 μm ID, 100 μm contact width, 4 mm spacing; Chávez Cerda et al., 2022) was placed around the nerve and sealed with surgical silicone. Electrodes were impedance-tested (< 20 kΩ) before implantation. VNA signal quality was confirmed postoperatively via detection of respiration and cardiac-related bursts (Stumpp et al., 2020). Leads were subcutaneously tunneled to the skull and fixed with UV dental cement. Postoperative care included twice-daily Tradamol^®^ and Ketofen^®^ (5 mg/kg each, s.c.) for 3 days, along with dietary enrichment to support recovery.

### 2.4 Signals recording and processing

Each rat was placed in a custom-built chronic monitoring system inside a Faraday cage to record seizures and VNA (Chávez Cerda et al., 2022). The setup enables free movement via slipring and allows real-time acquisition of VNA recordings, EEG, and video. Signals were amplified (10 MΩ input impedances, 800 V/V gain) with low noise (0.5 μV_RMS_) and filtered (10 Hz–10 kHz for VNA and 0.1–100 Hz for EEG). Recordings enabled time-aligned acquisition including two VNA channels (20 kS/s sampling rate), two EEG channels (1 kS/s sampling rate), and video (20 fps, Night-Vision Infrared camera, 5-megapixel). In preprocessing, VNA was bandpass-filtered to 300 Hz–3 kHz and EEG to 0.5–70 Hz. The system was previously validated (Chávez Cerda et al., 2022; Smets et al., 2022; Stumpp et al., 2020).

### 2.5 EEG analysis

Absence seizure onset and offset were identified EEG by characteristic 7–11 Hz slow wave discharge (300–1,000 μV) (Marescaux et al., 1992). We analyzed age-related seizure features in GAERS rats including seizure count per 24 h, duration, and ictal spiking frequency. To assess potential differences at seizure onset and offset spiking frequency was extracted via Fourier transform over the full seizure and the first and last 2 s segments.

### 2.6 VNA analysis

To analyze VNA changes during seizures, only absences ≥ 5 s and preceded by non-rapid eye movement (NREM) (0.5–4.0 Hz) were included to avoid movement artifacts. Even minor movements during wakefulness, such as grooming or postural adjustments, can introduce significant artifacts in recordings obtained from cuff electrodes. Therefore, to ensure optimal signal quality, we included only those seizures that were preceded by non-rapid eye movement (NREM) sleep in our analysis. For each event, a VNA segment matching the seizure duration and a clean 10 s pre-ictal baseline were extracted. RMS values were computed for both segments, and a VNA ratio (ictal RMS/baseline RMS) was calculated per seizure.

### 2.7 Statistical analyses

Statistical analyses were performed with GraphPad. Normality was assessed using the Shapiro-Wilcoxon test. Descriptive statistics (mean, SD, SEM) were reported for seizure features across age groups. Group differences were analyzed using the Kruskal-Wallis test with Dunn’s *post hoc* correction. RMS VNA ratios (median, 25th–75th percentiles) were compared similarly. Significance was set at α < 0.0001 (****). Interquartile ranges were compared using one-way ANOVA with Tukey’s test.

## 3 Results

### 3.1 Epilepsy characterization through age

The mean daily seizure counts (340, 339, and 324 for 4, 6, and 10 months-old rats, respectively) and seizure duration were similar across age groups (Table 1). Mean discharge frequencies during the entire seizure and the initial, and final 2 s of SWD also showed no significant differences (Table 1 and Supplementary Table 1).

**TABLE 1:** Absence seizure metrics by age.

Age	4 months				6 months				10 months			
Mean seizure/day ± sd	340 ± 60				339 ± 138				324 ± 79			
	Mean	Min	Max	SD	Mean	Min	Max	SD	Mean	Min	Max	SD
Seizure duration (sec)	**17.14**	2.59	161.70	±14.04	**15.79**	2.52	109.90	±15.79	**15.54**	1.50	158.30	±15.54
Whole SWD frequency (Hz)	**6.71**	4.15	8.30	±0.46	**6.32**	4.03	8.38	±0.62	**6.80**	4.03	10.25	±0.66
First 2 s SWD frequency (Hz)	**7.66**	5.86	11.72	±0.94	**7.21**	5.86	11.72	±1.09	**7.52**	5.86	11.72	±0.89
Last 2 s SWD frequency (Hz)	**6.54**	5.86	11.72	±1.25	**6.20**	5.86	11.72	±1.14	**6.50**	5.86	11.72	±0.95

Summary of 7,960 SWDs recorded over 48 h EEG recording in 12 animals, showing seizure and SWD frequency of (first 2 s, last 2 s) by age groups. Mean, minimum, maximum values, and standard deviations are reported. The bold values indicate the mean values per age.

### 3.2 VNA modulation during absences

Twelve rats were recorded, but one 10 months-old rat was excluded due to poor-quality VNA leaving 620 seizures from eleven rats for analysis.

**FIGURE 1:**
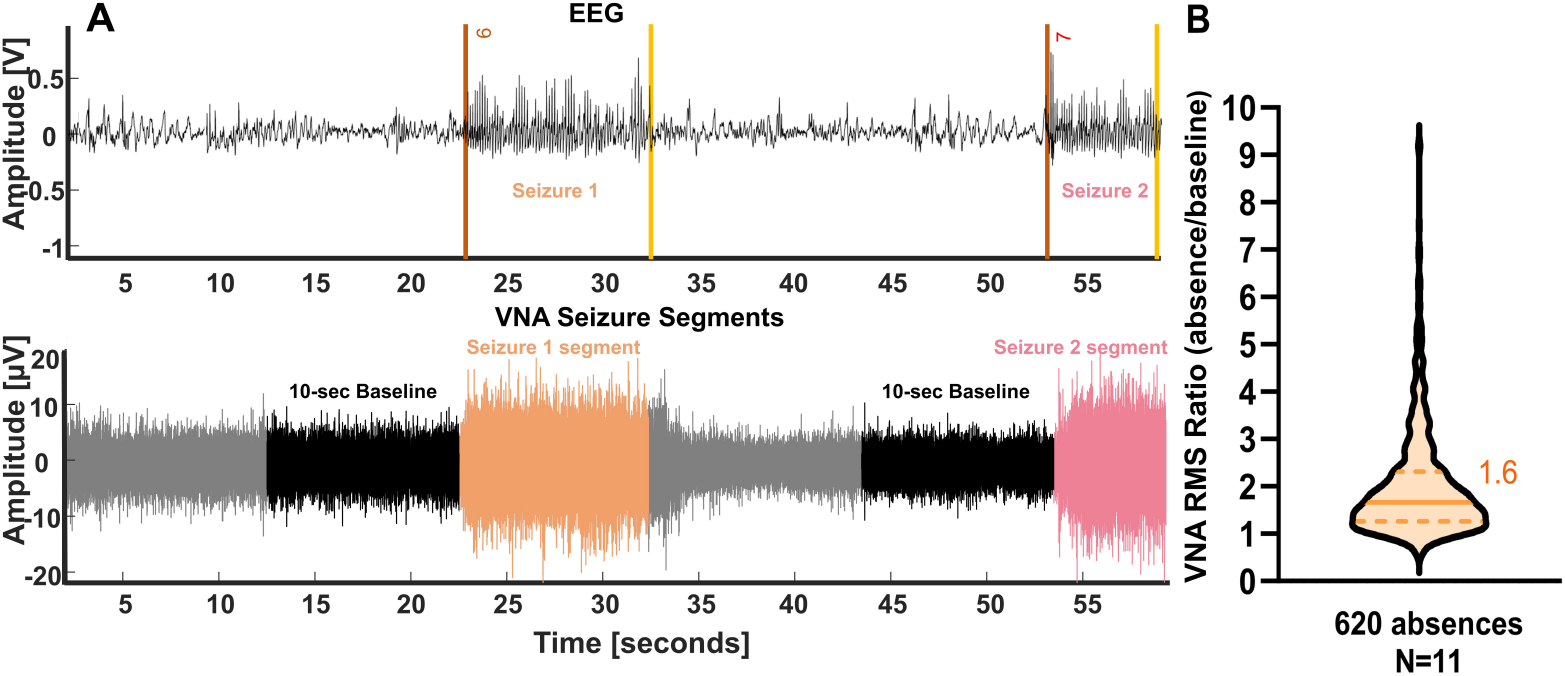
Vagus nerve activity is modulated during absences in Genetic Absence Epilepsy Rat from Strasbourg (GAERS) rats. **(A)** Example of vagus nerve activity increase during absence in one GAERS rat. Upper trace: bilateral scalp EEG recording. The onsets of the absences are shown by a red line and the offsets by a yellow line. Lower trace: bipolar vagus nerve activity (VNA) recording. The color of the vagus nerve activity corresponds to the color of the seizures detected on the EEG. The black segment is the pre-ictal phase selected as a 10 s baseline. **(B)** The root mean square (RMS) of the vagus nerve increases during SWDs compared to baseline. A median ratio of 1.6 is presented with a line, and the quartiles are shown in dashed lines (*n* = 620 seizures).

Figure 1A illustrates two absence seizures during NREM sleep with co-occurrence increases in VNA (orange and pink) compared to a 10 s baseline. Figure 1B displays RMS ratios indicating a median ratio of 60% VNA increase during seizures. Over 80% of seizures showed at least 20% increase, and 44% exceeded 80% (Supplementary Figure 1).

### 3.3 Effect of age on ictal VNA

**FIGURE 2:**
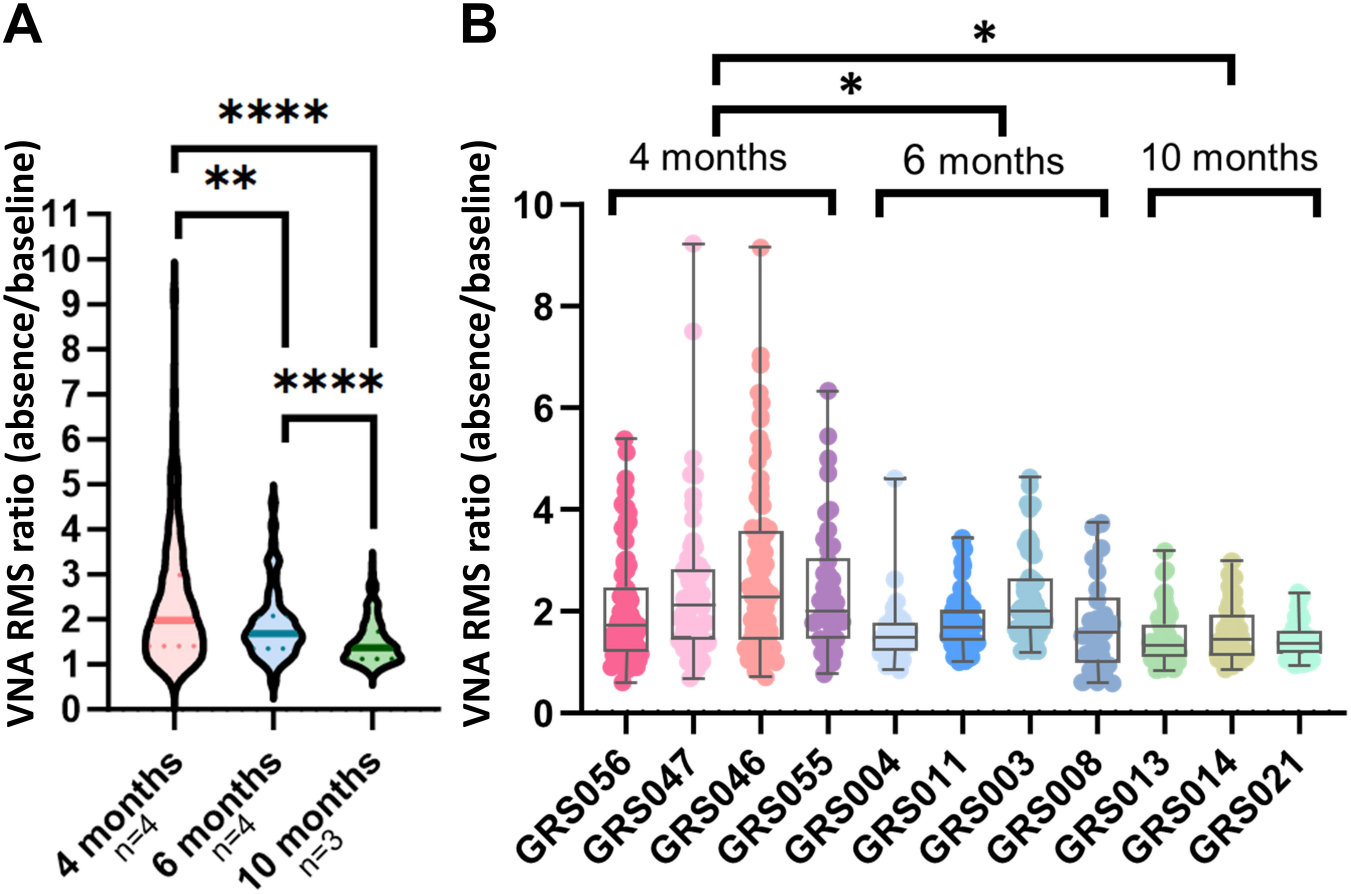
Ictal vagus nerve modulation decreases with age. **(A)** The root mean square (RMS) ratio of each seizure detected is presented pooled per age group. The line for each violin represents the median value. The dashed lines represent quartiles. Vagus nerve activity during absences in Genetic Absence Epilepsy Rat from Strasbourg (GAERS) decreases with age. **(B)** The dots representing vagus nerve modulation for each seizure per animal show a larger interquartile range between seizures in the 4 months-old rats compared to 6 and 10 months-old rats. The quartiles and the median are represented by a boxplot for each animal. The min and max values are shown with whiskers. *p < 0.05, **p < 0.01, ***p < 0.001.

To assess the effect of epilepsy duration on VNA, VNA recordings of eleven rats aged 4, 6, and 10 months were analyzed. Figure 2A depicts that VNA during seizures was highest 4 months-old rats c with median RMS ratio of 1.98, 1.68, and 1.37 for 4, 6, and 10 months-old GAERS, respectively (Supplementary Table 2). All age comparisons showed significant differences (Supplementary Table 3). Figure 2B reveals significant higher interquartile range in younger rats at 4 months compared to 6 and 10 months (*p*-value: 0.0352 and 0.0132) (Supplementary Table 4).

## 4 Discussion

This study demonstrates the feasibility of capturing VNA modulations during absences under chronic monitoring conditions, potentially offering new biomarkers for seizure during seizures to pre-ictal baselines during NREM sleep across three distinct age groups (4, 6, and 10 months). Our results show that VNA increases during absences, with a total mean increase of 60% in 620 seizures from eleven rats. Additionally, our findings revealed a significant age-dependent reduction in VNA during absences, while no discernible differences were observed in electrographic seizure characteristics among the three age groups.

Vagal visceral afferents play a significant role in epilepsy and autonomic alterations, as they convey visceral sensations from internal organs, including upper and lower airways, aortic baro- and chemoreceptors, to the central nervous system (Câmara and Griessenauer, 2015; Ruffoli et al., 2011). In healthy rats and humans, vagus nerve recordings have demonstrated that respiratory and ECG-related burst activity arises from both afferent and efferent components (Macefield et al., 2023; Ottaviani et al., 2020; Sevcencu et al., 2018). Physiological information such as blood pressure, heart, and respiration rate, or physiological behaviors in rats like eating and grooming were already extracted from the VNA (Marmerstein et al., 2022; Ottaviani et al., 2020; Stumpp et al., 2020). It could, therefore, be hypothesized that measuring vagus nerve activity might serve as a biomarker for dysregulation of the autonomic system in certain pathologies. In the context of epilepsy, Harreby et al. demonstrated that during induced pentylenetetrazol seizures under anesthesia, the cardiac-related vagus nerve signal exhibited significant ictal and post-ictal alterations (Harreby et al., 2011). In the same model, spike clustering detection revealed abrupt changes in VNA amplitude and frequency during induced seizures (Stumpp et al., 2021) and enabled earlier seizure detection compared to heart rate-based seizure detection methods (Stumpp et al., 2021). In the current study, the observed VNA augmentation during spontaneous seizures across all animals is coherent with the literature and encourages further research to explore the potential of VNA-based detection using signals obtained under chronic conditions.

Although ictal VNA generally increases, the patterns vary both across and within animals. In 6% of seizures, VNA RMS decreased compared to baseline; in 14%, it showed a slight increase (up to 20%); 80% of seizures showed an increase greater than 20%, and in 44% of cases, the rise exceeded 80%. While autonomic homeostasis relies on parasympathetic and sympathetic balance, seizures often disrupt this equilibrium, triggering autonomic symptoms across cardiovascular, respiratory, gastrointestinal, cutaneous, pupillary, genital, sexual, and urinary domains (Baumgartner et al., 2001). Sakamoto et al. (2008) showed that kainic acid-induced seizures in anesthetized rats increase both parasympathetic and sympathetic activities and impair baroreflex function with altered c-Fos expression in medullary regions. One plausible hypothesis to account for the observed heterogeneity in parasympathetic responses may stem from differences in seizure severity, both within individual animals and across different age cohorts. Severe seizures could induce more significant sympathetic activation and subsequent parasympathetic compensation (Naggar et al., 2022), while milder seizures might necessitate less pronounced restorative efforts. Although all seizures began during NREM sleep, post-ictal states varied, with possible transitions to wakefulness, activity, or REM sleep. This variability may have influenced ictal VNA modulation. However, without EMG data, post-ictal states could not be reliably classified.

Unlike Wag/Rij rats, which display two types of SWDs with type II concentrated in the occipital cortex (Sitnikova and van Luijtelaar, 2007), GAERS seizures after 4 months show consistent electrophysiological and behavioral features. Seizures are driven thalamocortically, originate in the primary somatosensory cortex, and are secondly generalized with stable frequency and duration from 4 months onward (Depaulis et al., 2016). Therefore, seizure severity likely doesn’t explain parasympathetic variability, though differences in onset may contribute. Most GAERS SWDs (91.9%) begin in the primary somatosensory cortex S1 with a preference for more anterior sites, while 8.1% begin simultaneously across cortical sites (Depaulis et al., 2016).

However, it is imperative to note that all seizures examined for VNA modulation were prefaced by a non-rapid eye movement (NREM) sleep period. Consequently, further investigations are warranted to validate the observed increase in VNA activity during absence seizures occurring throughout various phases of the day. In addition, a deeper understanding of VNA modulation in response to common physiological events alongside the progression of epilepsy could significantly enhance the development of reliable VNA-based seizure detection algorithms.

Altered vagus-mediated heart rate variability, together with increased epilepsy severity, is linked to higher SUDEP risk (DeGiorgio et al., 2010). Studies in children with drug-resistant epilepsy showed that non-treated epilepsy increases the risk of comorbidities and mortality due to a cardio-autonomic imbalance and left ventricular dysfunction (Ibrahim et al., 2021; Sakamoto et al., 2008). To explore autonomic alterations in epilepsy, we compared parasympathetic responses during seizures across age groups. In our study, no differences in seizure characteristics were found between age groups, but the median VNA RMS value during absence seizures was significantly lower in 10 months-old rats compared to 4 and 6 months-olds. This suggests that VNA response recorded during absence seizures diminishes with age, consistent with the notion that untreated epilepsy reduces parasympathetic tone (Yuen and Sander, 2017) and increases the risk of comorbidities and mortality due to cardio-autonomic and subclinical left ventricular dysfunction, independent of epilepsy duration, frequency, and seizure type (Ibrahim et al., 2021). However, the age-related changes in vagus nerve activity observed may also be influenced by age-dependent shifts in sympathetic and parasympathetic balance seen in healthy subjects (Pfeifer et al., 1983).

Lastly, we demonstrated that ictal parasympathetic inter-quartile range diminishes with age in GAERS rats, which might suggest reduced vagal responsiveness to sympathetic imbalance. This may reflect a cumulative strain on the parasympathetic system from repeated seizures leading to functional fatigue. Similar findings in WAG/Rij showed age-related reduction in HRV and increased sympathetic dominance (Mamalyga and Mamalyga, 2019). Vagus nerve activity is also subject to physiological modulation during natural behaviors such as feeding (Marmerstein et al., 2022) and may be different according to day/night (Smets et al., 2022). Thus, additional autonomic measures, such as electrocardiograms (ECG), heart rate variability (HRV), or respiratory rate concurrently recorded, could help distinguish seizure-related VNA changes from those driven by broader autonomic fluctuations.

We used an external cuff recording method to enhance translational relevance, though no control electrode devoid of vagus nerve input was included in our measurements. Consequently, it cannot be excluded that the recorded VNA signal may comprise a mixture of physiological and non-physiological signals. However, the potential effects of movement were minimized by focusing exclusively on absence seizures preceded by NREM sleep phases. For broader application to 24 h recording in freely moving animals, additional controls such as electromyography (EMG) and non-vagal contact are needed to isolate true vagus nerve activity, particularly during movement and to generalize the methodology to other epileptic models.

## 5 Conclusion

This study demonstrates for the first time that recording seizure-related modulations of VNA in chronic conditions in rats is possible. Those observations not only provide general insights into altered vagus nerve activity, as assessed by VNA recording, in relation to seizures, but also encourage further research into the potential of VNA-based detection using signals collected under chronic conditions. Consequently, it could pave the way for future studies measuring vagus nerve activity as a biomarker for autonomic system dysregulation in various pathologies.

## Data Availability

The data that support the findings of this study are not publicly available due to privacy reasons but are available from the corresponding author upon request and with the permission of Université Catholique de Louvain.
