# Supplementary material for: Vagal nerve signals are modulated by spontaneous seizures in Genetic Absence Epilepsy Rats from Strasbourg
Source: Front Neurosci. 2025 Jul 7;19:1568261. doi: 10.3389/fnins.2025.1568261 (PMC12277265; doi:10.3389/fnins.2025.1568261)
Supplement: Supplementary file 1 [file Data_Sheet_1.docx]

**Supplementary Material**

| **Kruskal Wallis test** |  |  |
| --- | --- | --- |
| **On seizure duration means** | Adjusted *ρ*-value | Significant? |
| 4 months vs. 6 months | 0.5094 | ns |
| 4 months vs. 10 months | 0.9804 | ns |
| 6 months vs. 10 months | >0.9999 | ns |
|  |  |  |
| **On whole seizure frequency** | Adjusted ρ-value | Significant? |
| 4 months vs. 6 months | 0.424 | ns |
| 4 months vs. 10 months | >0.9999 | ns |
| 6 months vs. 10 months | 0.0558 | ns |
|  |  |  |
| **On seizure frequency (2fisrt sec)** | Adjusted ρ-value | Significant? |
| 4 months vs. 6 months | 0.6072 | ns |
| 4 months vs. 10 months | >0.9999 | ns |
| 6 months vs. 10 months | >0.9999 | ns |
|  |  |  |
| **On seizure frequency (2last sec)** | Adjusted ρ-value | Significant? |
| 4 months vs. 6 months | 0.8422 | ns |
| 4 months vs. 10 months | >0.9999 | ns |
| 6 months vs. 10 months | 0.9804 | ns |

**Online Resource 1**. **Absence electrophysiology across age groups does not vary.** VNA ratio means value of each rat was computed and grouped by age. The Kruskal Wallis test has shown no significant differences between age groups for seizure duration, seizure frequency on the entire duration of each episode, or two first or last second of the seizure.


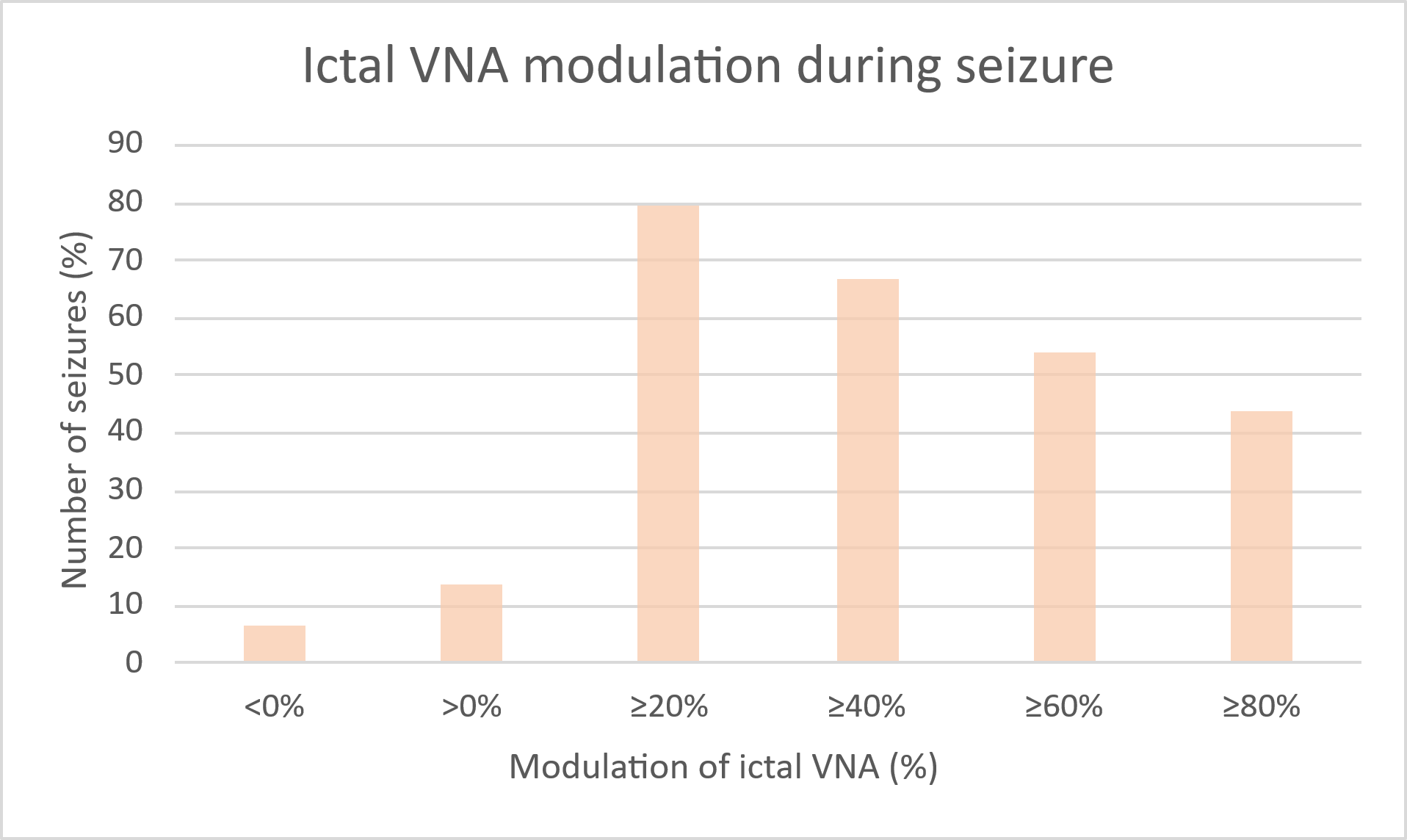


**Online Resource 2.** **An overview of VNA modulation during seizures in GAERS.** The histogram depicts the distribution of seizures characterized by ratios of 1.2, 1.4, 1.6, and 1.8 or higher was generated. Additionally, seizures with ratio of 1 or lower were included in the visualization. 0% VNA modulation means that no RMS difference was found between the seizure and 10-second baseline. <0% VNA modulation regroups the seizures with a decreased RMS compared to baseline.

|  | | |  |  |  |  |  |  |  |  | |  | |  |  |  | |  |
| --- | --- | --- | --- | --- | --- | --- | --- | --- | --- | --- | --- | --- | --- | --- | --- | --- | --- | --- |
|  | **4 months** | | | |  | **6 months** | | | | |  | | **10 months** | | | |  | |
|  | Rat1 | Rat 2 | Rat3 | Rat4 | **Grouped** | Rat5 | Rat6 | Rat7 | Rat8 | **Grouped** | | Rat9 | | Rat10 | Rat11 | **Grouped** | |  |
|  |  |  |  |  |  |  |  |  |  |  | |  | |  |  |  | |  |
| Minimum | 0.7059 | 0.6759 | 0.7722 | 0.5998 | 0.6 | 1.23 | 0.8491 | 0.5817 | 1.011 | 0.58 | | 0.8395 | | 0.8556 | 0.9347 | 0.84 | |  |
| 1st quartile | 1.441 | 1.466 | 1.468 | 1.202 | 1.405 | 1.654 | 1.219 | 0.9987 | 1.418 | 1.355 | | 1.109 | | 1.133 | 1.17 | 1.12 | |  |
| Median | 2.281 | 2.118 | 1.975 | 1.724 | **1.98** | 2.008 | 1.481 | 1.59 | 1.672 | **1.68** | | 1.322 | | 1.441 | 1.38 | **1.37** | |  |
| 3rd quartile | 3.575 | 2.817 | 3.032 | 2.469 | 2.99 | 2.647 | 1.759 | 2.274 | 2.015 | 2.08 | | 1.732 | | 1.93 | 1.608 | 1.73 | |  |
| Maximum | 9.152 | 9.218 | 6.326 | 5.386 | 9.22 | 4.629 | 4.615 | 3.732 | 3.441 | 4.63 | | 3.184 | | 2.985 | 2.375 | 3.18 | |  |
| **IQR** | **8.446** | **8.543** | **5.554** | **4.786** | **8.62** | **3.399** | **3.765** | **3.15** | **2.43** | **4.05** | | **2.345** | | **2.129** | **1.44** | **2.35** | |  |
|  |  |  |  |  |  |  |  |  |  |  | |  | |  |  |  | |  |
| Mean | 2.778 | 2.441 | 2.404 | 2.038 | **2.42** | 2.299 | 1.573 | 1.73 | 1.775 | **1.86** | | 1.455 | | 1.585 | 1.442 | **1.5** | |  |
| Std. Deviation | 1.722 | 1.497 | 1.272 | 1.104 | **1.46** | 0.8955 | 0.6441 | 0.9269 | 0.5562 | **0.77** | | 0.4907 | | 0.5381 | 0.368 | **0.48** | |  |
| Std. Error of Mean | 0.1902 | 0.1933 | 0.1963 | 0.1234 | 0.09 | 0.1279 | 0.1074 | 0.1818 | 0.0638 | 0.06 | | 0.06087 | | 0.0678 | 0.05747 | 0.04 | |  |

**Online Resource 3. Descriptive statistics of ictal vagus nerve modulation per age group.** Analysis was done on 620 seizures divided by age: 4 months (n=4), 6 months (n=4), and 10 months (n=3). The minimum and maximum values are shown per age group, as well as the median and the two quartiles and the inter-quartile range (IQR). The range of values, the mean, the standard deviation, and the error of mean are presented. The columns shaded in gray represent all ictal VNA modulation values aggregated for each age group

|  |  |  |  |
| --- | --- | --- | --- |
| **Dunn's multiple comparisons test** | Adjusted ρ-value | Summary | Significant? |
| 4 months vs. 6 months | 0.0077 | ** | Yes |
| 4 months vs. 10 months | <0.0001 | **** | Yes |
| 6 months vs. 10 months | <0.0001 | **** | Yes |

| **Tukey's multiple comparisons test** | Adjusted ρ-value | Summary | Significant? |
| --- | --- | --- | --- |
| 4 months vs. 6 months | 0.035 | * | Yes |
| 4 months vs. 10 months | 0.0132 | * | Yes |
| 6 months vs. 10 months | 0.6472 | ns | No |
|  |  |  |  |

**Online Resource 4. Median VNA RMS ratios differ between age groups: a Dunn multiple comparison test**. Each median was calculated by plotting all RMS values from seizures of rats aged 4, 6, and 10 months.

**Online Resource 5. VNA modulation variability decreases with age: interquartile range comparison.** Interquartile range (IQR) values were calculated for each rat. A Turkey’s multiple comparison test compared IQR values between ages and showed significant differences between 4 and 6 months as well as between the 4- and 10-months rats.
